# Relationship between Diet, Menstrual Pain and other Menstrual Characteristics among Spanish Students

**DOI:** 10.3390/nu12061759

**Published:** 2020-06-12

**Authors:** María Dolores Onieva-Zafra, Elia Fernández-Martínez, Ana Abreu-Sánchez, María Teresa Iglesias-López, Francisca María García-Padilla, Miguel Pedregal-González, María Laura Parra-Fernández

**Affiliations:** 1 Department of Nursing, Physiotherapy and Occupational Therapy, University of Castilla-La-Mancha, Ciudad Real, 13071 Ciudad Real, Spain; mariadolores.onieva@uclm.es (M.D.O.-Z.); marialaura.parra@uclm.es (M.L.P.-F.); 2 Department of Nursing, University of Huelva, 21004 Huelva, Spain; abreu@denf.uhu.es (A.A.-S.); fmgarcia@denf.uhu.es (F.M.G.-P.); miguel.pedregal@denf.uhu.es (M.P.-G.); 3 Faculty of Health Sciences, Universidad Francisco de Vitoria, Crta. Pozuelo-Majadahonda km 1,800, 28223 Pozuelo de Alarcón (Madrid), Spain; m.iglesias.prof@ufv.es; 4 Multiprofessional Unit of Family and Community Care of Huelva, 21007 Huelva, Spain

**Keywords:** Mediterranean diet, woman, menstrual disorder

## Abstract

This study sought to describe and compare adherence to the Mediterranean diet and consumption of local foods from the Huelva region among Spanish university women in relation to menstrual pain and other menstrual characteristics. This cross-sectional study included 311 health science students. The study variables were sociodemographic and gynecologic characteristics, adherence to the Mediterranean diet using the KIDMED questionnaire, alcohol consumption (SDU) and consumption of local food. A descriptive bivariate analysis and multiple binary regression were performed for menstrual pain. Up to 55.3% of participants had moderate adherence to the Mediterranean diet and only 29.6% had high adherence. Women with low adherence had longer menstrual cycles (*p* < 0.01). Eating less than two pieces of fruit per day (OR = 3.574; 95%CI = 1.474–8.665; *p* < 0.05) and eating pulses more than one day a week (OR = 2.320; 95%CI = 1.006–5.348) raised the probability of suffering menstrual pain. A positive correlation between SDU and cycle length was identified (*r* = 0.119, *p* = 0.038), and menstrual bleeding was lower in women who consumed olive oil daily (*p* = 0.044). In conclusion, the Mediterranean diet, alcohol consumption and consuming typical foods from southern Spain appear to influence cycle length, menstrual flow and menstrual pain. Further research is necessary to confirm and expand these findings.

## 1. Introduction

The Mediterranean diet (MD) is a traditional dietary pattern among Mediterranean countries that is characterized by a high consumption of fruits, vegetables, cereals, pulses, a moderate consumption of white meat, fish and alcohol and a low consumption of sugars and red and processed meats. It is also characterized by a low consumption of saturated fats and the use of olive oil as the main fat source [1]. Several studies have identified several health benefits in individuals with a high adherence to MD. These are mainly focused on cardiovascular aspects, cancer prevention, anti-inflammatory properties, a decreased mortality and an improved quality of life [1,2,3,4]. Indeed, the MD has been considered as an Intangible Cultural Heritage of Humanity by UNESCO (2010) [5]. The interest in MD also seems to be related to traditions, the accessibility of raw materials and having time to prepare healthy recipes [6]. However, in recent years in Spain, less adherence to this type of diet has been identified among young people, an aspect that is of concern due to its potential impact on their present and future health [7].

In women, the menstrual cycle begins at puberty and it is during these early years that women experience the most alterations of their menstrual cycle. A normal menstrual cycle is regular and lasts between 24 and 38 days. Once per cycle, women have their period, with menstrual flow that usually lasts from 5 to 8 days. The usual amount of blood loss is between 5 and 80 mL [8,9]. Menstrual pain or cramps, also known as dysmenorrhea, is a common problem related to the menstrual cycle in young women. It usually presents as chronic pelvic or lower abdominal pain. Sometimes, it is accompanied by several other symptoms, such as feelings of depression, dizziness, irritability, diarrhea or nausea. Most often, this problem is not associated with any organic cause, in which case it is known as primary dysmenorrhea [10,11,12]. Other frequent menstrual cycle problems in young adults are irregular cycles and irregularities in the amount of bleeding [13]. Other health disorders may also affect the menstrual cycle. This is the case of certain eating disorders which are related to irregularities and periods of amenorrhea [14,15].

Concerning dietary factors, several previous studies have analyzed the possible influence between the consumption of certain foods and menstrual pain, highlighting the potentially protective role of increased consumption of fruits, vegetables, fish and dairy products against menstrual pain; however, the evidence on this subject remains limited at present and further research is warranted [16,17]. Regarding the study of the MD and typical foods consumed in southern Spain in relation to the characteristics of the menstrual cycle and menstrual pain, no evidence is found in the literature. However, some studies conducted in other countries already point to the positive influence of the MD on other aspects of women’s health such as reproductive function [18,19] and a reduced risk of breast cancer [20]. Furthermore, according to certain studies, three foods typically consumed in the region of Huelva are described as having anti-inflammatory and antioxidant properties and are therefore considered potentially beneficial to overall health: concretely, strawberries, olive oil and Spanish ham [21,22,23,24]. The present study aims to describe and compare adherence to the MD and consumption of local foods in the region of Huelva among Spanish university women in relation to menstrual pain and other menstrual characteristics.

The hypotheses were:

- The adherence to the Mediterranean diet and the consumption of some local foods affect the characteristics of the menstrual cycle of young university women.
- Women who follow the recommendations of a Mediterranean diet and consume some of the typical local foods are less likely to suffer from menstrual pain

## 2. Materials and Methods

This is a cross-sectional descriptive study carried out in southern Spain on health science students at the University of Huelva, Andalusia. The inclusion criteria were women aged between 18 and 35 years old, enrolled in the Faculty of Nursing at the University of Huelva in the academic year 2018/2019 and who voluntarily accepted to participate in this study. Students who were abroad on an academic exchange at the time of data collection were excluded. Recruitment of the sample was performed by inviting all female health science students enrolled in the specified academic year to participate in the study. All the students agreed to participate and of these 94% met the inclusion criteria (in terms of age and not being on an exchange) and were therefore included in the study sample.

### 2.1. Data Collection

Data were collected using a self-report paper questionnaire. This questionnaire was purposely designed by the research team based on previous studies. During the classroom period, the participants were invited to participate in the study by a teacher at the University of Huelva. A researcher provided information about the study and was present while participants completed the questionnaire in order to clarify possible doubts. The questionnaire featured sociodemographic and gynecological questions. The intensity of self-reported menstrual pain was evaluated using the horizontal visual analogue scale (VAS) from 0 to 10 and was interpreted as in previous studies: mild (1–3), moderate (4–6) and severe (7–10) [12,25,26]. Regarding food consumption, three questions were included on the consumption of typical foods from the Andalusian region: olive oil, cured ham and strawberries. The KIDMED questionnaire was also included in its original Spanish version, which consists of 16 items on adherence to the MD [27,28]. Finally, we asked about daily consumption of alcoholic beverages by requesting the participant to indicate the standard drink units (SDU). This is an instrument for the standardized measurement of an individual’s daily consumption. The Spanish Scientific Society for the Study of Alcoholism and other Drug Addictions established that one SDU is equivalent to about 10 g of pure alcohol [29,30].

### 2.2. Data Analysis

The data were imported into an Excel spreadsheet of the Microsoft Office package and were subsequently analyzed using SPSS v23 Statistical Package for Social Sciences. For the descriptive analysis, frequencies and percentages were used for qualitative variables whereas for the quantitative variables, the mean and standard deviation were used.

Regarding the bivariate analyses, a chi-square test was used to compare qualitative variables among various groups, such as in women with and without menstrual pain and Student’s t-test was used to compare quantitative variables in the same groups. A chi-square linear trend test was used to compare the three groups of adherence to the MD and local food consumption compared to different menstrual characteristics. One-factor ANOVA of polynomial contrasts and post hoc tests were used to compare quantitative variables among the three groups of women according to their adherence to the MD. Correlation analyses were performed using the Pearson’s coefficient to seek linear correlations between KIDMED and SDU scores and quantitative gynecological variables. A multiple binary stepwise logistic regression model by age, BMI and use of hormonal contraceptives was used, in which the predictor variable was suffering from menstrual pain; also, the KIDMED questionnaire items, SDU and typical local food consumption were included. The appropriateness of conditions for the application of the statistical tests employed was verified in all cases. The significance level was set at *p* < 0.05.

### 2.3. Ethical Considerations

All students participated voluntarily after signing the informed consent. The project obtained a favorable report from the Andalusian Committee for Biomedical Research and the data were treated anonymously at all times and conducted according to the principles of the Declaration of Helsinki.

## 3. Results

### 3.1. Socio-Demographic Characteristics

In total, 311 women participated in this study, with a mean age of 21.17 ± 2.56 years, a mean height of 164.35 ± 6.22cm, a mean weight of 60.53 ± 9.48 kg and a BMI of 22.40 ± 3.17 m^2^/kg. According to the BMI classification of the World Health Organization (WHO), 5.5% were underweight, 78.8% were normal weight, 12.5% were overweight and 3.2% of participants were obese [31].

### 3.2. Adherence to Mediterranean Diet, Alcohol Consumption and Local Foods

The average score on the KIDMED Scale was 6.14 ± 2.39 for all participants. Up to 15.1% (47) had low adherence to the MD, 55.3% (172) had moderate adherence, and 29.6% (92) had high adherence. No differences were found when comparing adherence to the MD as a function of the sociodemographic variables analyzed.

The average alcohol consumption was 2.64 ± 3.43 SDU, with 0 SDU being the minimum consumption and 30 SDU the maximum self-reported consumption. Concerning the consumption of local food, 5.5% consumed strawberries daily and 88.4% consumed olive oil daily. Regarding the consumption of cured ham, 35.7% of the participants reported consuming it once a week.

### 3.3. Diet and Characteristics of the Menstrual Cycle

When analyzing the mean scores of the KIDMED questionnaire of adhesion to the MD and comparing this among women with irregular (6.20 ± 2.59) and regular (6.10 ± 2.30) cycles, no differences were found (*p* > 0.05). Furthermore, there was no correlation between the KIDMED score and cycle length (*r* = −0.066, *p* > 0.05), nor with the duration of menses (*r* = 0.029, *p* > 0.05). Regarding the amount of menstrual flow, a higher mean KIDMED score was found among women with heavy menstrual flow (6.86 ± 2.10) compared to those with a medium amount of flow (5.83 ± 2.43) (*p* < 0.01).

After grouping the participants into three categories according to the interpretation of the KIDMED, as described in the previous literature, and comparing their menstrual cycle characteristics (Table 1), statistically significant differences were only found for the length of the menstrual cycle, which was longer in women with low adherence to the MD (*p* < 0.01).

When analyzing alcohol consumption measured in SDU, along with menstrual characteristics, no differences were found in relation to regularity, amount of flow or duration of menses. A positive correlation was only found between SDU of alcohol consumption and cycle length (*r* = 0.119, *p* = 0.038).

Regarding the consumption of local food (ham, strawberry and olive oil) and the relationship with diet and the menstrual characteristics of women, statistically significant differences were only found when comparing the amount of menstrual flow of women who consumed olive oil daily and those who did not (*p* = 0.044). Thus, in women who consumed olive oil daily, a lower percentage of women were identified as having heavy bleeding (21.8%) versus 25% among women who did not consume olive oil. Regarding the weekly consumption of cured serrano ham, a greater number of women who consumed ham with this frequency reported heavy bleeding (30.6%) compared to those who did not (17.5%) (*p* ≤ 0.01).

### 3.4. Diet and Menstrual Pain

No difference in the mean KIDMED Scale score was found between women with menstrual pain (6.13 ± 2.38) and those without (6.17 ± 2.44) or when comparing groups with different MD adherence. In the item-by-item comparison of participants’ responses to the KIDMED questionnaire between women who suffered from menstrual pain and those who did not, statistically significant differences were only found in relation to Item 2 of the KIDMED questionnaire referring to fruit consumption (Table 2). More women without dysmenorrhea consumed a second piece of fruit compared to women with dysmenorrhea (*p* < 0.05). In the regression model, this item was identified as a protective factor for dysmenorrhea, observing that not consuming a second piece of fruit increased the probability of suffering this pain by 2.984 (95%CI = 1.390–6.406; *p* < 0.05). Item 7, which corresponded with “Likes pulses and eats them >1/week” was also identified as a risk factor, which increased this likelihood by 2.320 (95%CI = 1.006–5.348) times (Table 3). In relation to the consumption of typical local foods and menstrual pain, daily strawberry consumption among women without dysmenorrhea was higher (11.4%) than among those with dysmenorrhea (4.7%). The percentage of women who consumed olive oil daily was higher among those who did not suffer from dysmenorrhea (91.4%) than among those who did (88%), however this difference was not significant. The percentage of women who ate cured Serrano ham on a weekly basis was slightly higher but not significant in women who suffered from dysmenorrhea (35.9%) compared to those who did not (34.3%). Neither was there any difference in alcohol consumption measured in SDU between the two groups.

No significant differences were found when comparing the adherence to the MD among women with and without symptoms related to menstrual pain, i.e., tiredness, breastfeeding, nausea, vomiting, headache, diarrhea, irritability and feeling depressed during menstruation.

Of the 276 women suffering from menstrual pain, 273 provided information on the intensity of their pain according to the VAS Scale. No linear correlation was identified between pain intensity scores using the VAS and the MD adherence score measured using the KIDMED questionnaire (r= 0.035, *p* = 0.563). The mean intensity of menstrual pain was 6.83 ± 1.64 in the participants with dysmenorrhea. When comparing the mean intensity of menstrual pain on the VAS scale among women with varying degrees of adherence to the MD, no differences were found (Table 4).

When women were categorized in relation to the intensity of dysmenorrhea pain, 4% reported having mild pain, 30% had moderate pain and 65.9% had severe pain. Comparing these groups with the degrees of adherence to the MD, no differences were identified (Table 5).

When comparing each item of the KIDMED questionnaire among women according to pain intensity, no differences were found between groups (Table 6).

The average alcohol consumption among women with mild pain was 2.91 ± 3.39 SDU, for those with moderate pain, this was 2.77 ± 3.39 SDU and for those with severe pain alcohol consumption was 2.56 ± 3.54 SDU, with no significant differences found between these groups.

In relation to the regular intake of typical local foods, no differences were found in relation to the consumption of strawberries, neither were there differences in relation to the daily consumption of olive oil, nor in relation to the weekly consumption of Serrano ham. However, regarding the consumption of cured ham, we identified a greater percentage of women who consumed ham among those with severe pain (38.3%), compared to the percentage of women who consumed ham with moderate pain (31.7%) and mild pain (18.2%).

## 4. Discussion

This study described and compared adherence to the MD and consumption of local foods from the Huelva region among university women in relation to menstrual pain and other menstrual characteristics. Over half of participants were identified as having moderate adherence and only 29.6% had high adherence. Women with low adherence to the MD were found to have longer menstrual cycles, although no differences were found in suffering from menstrual pain among women with different levels of MD adherence. Nonetheless, we found that women who ate less than two pieces of fruit per day were 2.984 (95%CI = 1.390–6.406; *p* < 0.05) more likely to suffer menstrual pain, which was also the case of women who liked pulses and ate them >1/week, for whom the likelihood was 2.320 (95%CI = 1.006–5.348) greater. Concerning alcohol consumption in the young women consulted, the mean SDU was 2.64 ± 3.43 and a positive correlation was identified between SDU and cycle length. In relation to the consumption of typical products of the region, 5.5% consumed strawberries daily, 88.4% consumed olive oil daily and 35.7% ate cured ham on a weekly basis. The amount of menstrual bleeding was lighter in women who consumed olive oil daily. In contrast, it was heavier among those who consumed ham weekly. Although no statistically significant differences were found, the consumption of strawberries and olive oil daily was higher among women who did not suffer from menstrual pain compared to those who did, and the consumption and percentage of women who ate cured ham at least once a week was slightly higher in women who suffered from dysmenorrhea.

Our proportion of low adherence to the MD was slightly lower than the percentage reported in women in two previous recent studies on university students in central Spain, which identified adherence rates as low as 20% and 38.1% for this type of diet [32,33]. Regarding the rates for women with high adherence to the MD, they were higher than those identified in previous studies such as that of García-Meseguer et al. who identified a high adherence in only 6.9% of women [33]. These figures indicating greater adherence to MD can be attributed to the fact that in the southern region of Spain, where this study was carried out, there is a greater availability of typical products of the MD from local agriculture which may facilitate access to these products at an affordable cost. Nonetheless, a comparison of findings is complex as different instruments have been used to assess adherence to MD in previous studies.

Previous studies have reported multiple potential benefits of the MD for different health problems such as cardiovascular and oncological problems, relating this type of diet with a better quality of life. On the gynecological level, benefits have been studied in relation to breast cancer and fertility; however, the present study, in relation to menstrual characteristics, only identified a longer duration of the menstrual cycle and lighter periods in women with low adherence to the diet. No previous studies were found to compare this outcome, although some studies have identified platelet, inflammatory and hormonal influences on MD adherence [34], which are aspects that could be related to this finding. No differences were found between women with different levels of adherence to the MD and dysmenorrhea despite the fact that the anti-inflammatory potential of the MD has been shown to influence the prevention of other inflammatory processes such as rheumatoid arthritis [2,4]. However, a lower percentage of dysmenorrhea was found among those who met the MD criterion, which was the consumption of at least two pieces of fruit daily; this finding is consistent with several previous studies that attribute this to mineral and vitamin content [16,35,36]. More concretely, when analyzing the consumption of strawberries, a typical product of the region, no statistically significant differences were found. This may be attributed to the fact that this is a seasonal product and we asked students about their daily consumption at a time when the amount of fresh product available was very low and sold at the highest price. This was reflected in the fact that only 5.5% of the participants acknowledged consuming it daily, making it difficult to make a comparison between groups, according to the differences in menstrual characteristics. This opens an interesting line of research, since the amount of menstrual bleeding and the prevalence of menstrual pain were lower among women who consumed strawberries, considering that previous studies have demonstrated that consumption of strawberries has known antioxidant, anti-inflammatory and antihypertensive effects in both animals and humans [22]. Regarding the consumption of pulses more than once a week, which was identified as a potential risk factor for menstrual pain, this contrasts with traditional advice provided in Chinese cultures which recommend red bean soup for menstrual pain [37]. However, we were unable to identify any studies to analyze the effects of pulses on menstrual pain and therefore it would be interesting to continue exploring the effects of this food. Furthermore, in a study conducted by Abu Helwa et al., skipping breakfast was the strongest predictor of severity of dysmenorrhea [37]. However, in our study, no significance was found for this item of the KIDMED questionnaire in relation to the presence of dysmenorrhea or among women with different levels of menstrual pain. Nonetheless, it should be noted that in Spain, breakfast is a traditionally established meal and only 11.6% of our participants skipped breakfast.

In women with low adherence to the MD, a greater duration of the menstrual cycle was detected. This could be because the MD implies a lower consumption of meat than other diets, also considering that previous studies have shown that a high dietary intake of meat modifies the release of gonadotropin hormones and follicular maturation by increasing the duration of the cycle [38].

Previous studies have identified a possible influence of meat consumption in relation to the presence of dysmenorrhea, identifying a lower prevalence of this problem in vegetarian women and attributing this to a decrease in estrogenic activity due to the absence of meat intake [12,39,40]. Our study shows results along these lines, as the percentage of women who ate Serrano ham at least once a week was slightly higher in women who suffered from dysmenorrhea. Moreover, when analyzing all women who suffered from dysmenorrhea, more cases of severe intensity were found among those who reported consuming cured ham on a weekly basis. It should be noted that cured Serrano ham is a type of salted, processed red meat, which is traditionally consumed in Spain and increasingly in other European countries. However, the World Health Organization (WHO) considers the consumption of processed meat as unhealthy due to its harmful effects on health, mainly oncological and cardiovascular, and therefore, in general, consumption should be limited [41,42]. Despite this, the consumption of cured ham should continue to be studied specifically in relation to various aspects of health, as there are studies that indicate that this consumption does not have the same harmful effects on health as those attributed to the consumption of other processed red meat. For example, cured ham is considered a natural source of bioactive peptides with antihypertensive and antioxidant effects that are beneficial to health [43,44]

No association was found between alcohol consumption and dysmenorrhea; this finding is consistent with the results of different previous studies conducted in different countries [12,45,46]. However, a positive correlation was found between alcohol SDU and menstrual cycle length. This finding is consistent with a study conducted by J Lyngsø et al. in which they also identified shorter cycles among women abstaining from alcohol, associating these alterations with the fact that alcohol exposure can produce alterations in the hormonal system [47]. In addition, studies in rats have associated chronic alcohol consumption with endometrial changes and reproductive disorders [48].

Previous studies have identified that fish oils may present protective factors against menstrual pain [49,50]. In line with our results, a previous study carried out among Spanish university women identified a lower proportion of women with primary dysmenorrhea among those who always cooked with olive oil [12]. The possible effects of these oils as potential protectors against this gynecological problem are attributed to their anti-inflammatory properties and their possible influence on the physiopathological mechanism responsible for most of the menstrual pain experienced by young women, which is sustained by the increased levels of prostaglandins [24]. Regarding the finding that menstrual flow was lighter in women who consumed olive oil daily, we were unable to find any studies with which to compare these findings. Therefore, it would be interesting to continue to study this line of research in relation to the relevance of the prevention of large amounts of blood loss in menstruation due to the associated risk of anemia. Heavy menstrual bleeding causes iron deficiency anemia and also conditions normal activity among more than half of the young women who suffer it [51,52].

The present study stands out for being the first to jointly study the MD and the consumption of typical products from the south of Spain in relation to the menstrual characteristics of young people. However, these results should be interpreted with caution, as the data were obtained from a cross-sectional study by means of self-reporting. Furthermore, the study sample only included women from a single university in southern Spain. In addition, the instrument used to evaluate adherence to MD, KIDMED, was developed for people from 2–24 years old, and therefore it was considered ideal for evaluating 91.3% of the participants. However, it was also used to evaluate diet in women over 24 years of age (8.7% of the sample). Nonetheless, we were able to find other studies which also employed this scale among university students [53,54]. The findings of the present study open a new line of research on the possible influence of the MD and the consumption of foods that are typical of certain regions, in relation to menstrual characteristics.

## 5. Conclusions

Most university women have a moderate adherence to the MD. Menstrual pain was associated with eating less than two portions of fruit per day. High and moderate adherence to the Mediterranean diet and lower alcohol consumption appear to be related to shorter cycles. Regarding consuming typical foods from southern Spain, we identified that women who consumed olive oil had less daily menstrual bleeding. Although many studies have previously analyzed the consumption of some foods in relation to menstrual pain, this study is a first approximation to the adherence to the MD, the consumption of typical food from the south of Spain and its possible influence on the characteristics of the female menstrual cycle and menstrual pain. Further studies are warranted to confirm and extend these findings.

## Figures and Tables

**Table 1:** Menstrual characteristics and adherence to the Mediterranean diet (MD).

		Adherence to the MD			*p*-Value
		Low	Average	High	
Cycle	Regular	19(40.4%)	42(24.4%)	32(34.8%)	0.931 ^a^
	Irregular	28(59.6%)	130(75.6%)	60(65.2%)	
Amount of flow	Light	9(19.1%)	27(15.7%)	19(20.7%)	0.337 ^a^
	Average	35(74.5%)	101(58.7%)	51(55.4%)	
	Heavy	3(6.4%)	44(25.6%)	22(23.9%)	
Duration of cycle		33.47 ± 16.23	29.15 ± 5.74	30.16 ± 6.28	0.008 ^b,^*
Duration of menses (days)		5.17 ± 1.22	4.87 ± 1.21	5.01 ± 1.31	0.308 ^b^
Menstrual pain	No	6(12.8%)	21(12.2%)	35(11.3%)	0.400 ^a^
	Yes	41(87.2%)	151(87.8%)	276(88.7%)	

*p < 0.05; ^a^ Chi-square for linear trend; ^b^ ANOVA of polynomial contrasts.

**Table 2:** Comparison of responses to items on the KIDMED questionnaire between participants with and without dysmenorrhea.

		Menstrual Pain		Total	*p*-Value
		No (*n* = 35)	Yes (*n* = 276)		
Kidmed1. Has a fruit or natural fruit juice every day.	No	14(40%)	115(41.7%)	129(41.5%)	0.850
	Yes	21(60%)	161(58.3%)	182(58.5%)	
Kidmed2. Has a second piece of fruit every day.	No	18(51.4%)	198(71.7%)	216(69.5%)	0.014 *
	Yes	17(48.6%)	78(28.3%)	95(30.5%)	
Kidmed3. Has fresh or cooked vegetables regularly once a day.	No	10(28.6%)	86(31.2%)	96(30.9%)	0.755
	Yes	25(71.4%)	190(68.8%)	215(69.1%)	
Kidmed4. Has fresh or cooked vegetables regularly more than once a day.	No	25(71.4%)	184(66.7%)	209(67.2%)	0.572
	Yes	10(28.6%)	92(33.3%)	102(32.8%)	
Kidmed5. Consumes fish regularly (at least 2–3/week).	No	18(51.4%)	112(40.6%)	130(41.8%)	0.220
	Yes	17(48.6%)	164(59.4%)	181(58.2%)	
Kidmed6. Goes >1/week to a fast food restaurant (hamburger chain).	No	25(71.4%)	202(73.2%)	227(73%)	0.825
	Yes	10(28.6%)	74(26.8%)	84(27%)	
Kidmed7. Likes pulses and eats them >1/week.	No	11(31.4%)	55(19.9%)	66(21.2%)	0.117
	Yes	24(68.6%)	221(80.1%)	245(78.8%)	
Kidmed8. Consumes pasta or rice almost every day (5 days or more per week).	No	25(71.4%)	218(79%)	243(78.1%)	0.308
	Yes	10(28.6%)	58(21%)	68(21.9%)	
Kidmed9. Has cereal or grains (bread, etc.) for breakfast.	No	10(28.6%)	60(21.7%)	70(22.5%)	0.362
	Yes	25(71.4%)	216(78.3%)	241(77.5%)	
Kidmed10. Has cereal or grains (bread, etc.) for breakfast.	No	15(42.9%)	154(55.8%)	169(54.3%)	0.148
	Yes	20(57.1%)	122(44.2%)	142(45.7%)	
Kidmed11. Uses olive oil at home.	No	1(2.9%)	5(1.8%)	6(1.9%)	0.672
	Yes	34(97.1%)	271(98.2%)	305(98.1%)	
Kidmed12. Skips breakfast.	No	31(88.6%)	244(88.4%)	275(88.4%)	0.997
	Yes	4(11.4%)	32(11.6%)	36(11.6%)	
Kidmed13. Has a dairy product for breakfast (yogurt, milk, etc.).	No	13(37.1%)	108(39.1%)	121(38.9%)	0.820
	Yes	22(62.9%)	168(60.9%)	190(61.1%)	
Kidmed14. Has commercially baked goods or pastries for breakfast.	No	31(88.6%)	260(94.2%)	291(93.6%)	0.201
	Yes	4(11.4%)	16(5.8%)	20(6.4%)	
Kidmed15. Has 2 yoghurts and/or 40 g of cheese daily.	No	24(68.6%)	189(68.5%)	213(68.5%)	0.991
	Yes	11(31.4%)	87(31.5%)	98(31.5%)	
Kidmed16. Eats sweets and/or candy several times a day.	No	33(94.3%)	262(94.9%)	295(94.9%)	0.871
	Yes	2(5.7%)	14(5.1%)	16(5.1%)	

*p < 0.05.

**Table 3:** Multiple stepwise regression to predict menstrual pain based on diet.

	OR^a^	CI(95%)
Item2 KIDMED	0.335 *	0.156–0.720
Item7 KIDMED	2.320 *	1.006–5.348

OR^a^: Odds ratio adjusted by age, BMI and hormonal contraceptives; *p < 0.05.

**Table 4:** Comparison of the mean intensity of menstrual pain (VAS) among women with different levels of MD adherence.

Levels of MD Adherence	Intensity of Menstrual Pain (VAS)	*p*-Value ^a^
	Mean ± SD	
Low adherence	6.77 ± 1.65	0.671
Average adherence	6.91 ± 1.55	
High adherence	6.83 ± 1.64	

^a^ ANOVA of polynomial contrasts.

**Table 5:** Comparison of proportions of women grouped by MD adherence levels and menstrual pain intensity categories.

		Pain Intensity (VAS)			Total	*p*-Value ^a^
		Mild	Moderate	Severe		
**Adherence to a MD**	**Low**	2(5.1%)	13(33.3%)	24(61.5%)	39(14.3%)	0.887
	**Average**	5(3.3%)	42(28%)	103(68.7%)	150(54.9%)	
	**High**	4(4.8%)	27(32.1%)	53(63.1%)	84(30.8%)	

^a^ Chi-square for linear trend.

**Table 6:** Comparison of women grouped according to pain intensity and MD adherence based on KIDMED questionnaire items.

		Pain Intensity (VAS)			Total	*p*-Value ^a^
		Mild	Moderate	Severe		
Kidmed1. Has a fruit or natural fruit juice every day.	No	4(36.4%)	36(43.9%)	73(40.6%)	113(41.4%)	0.835
	Yes	7(63.6%)	46(56.1%)	107(59.4%)	160(58.6%)	
Kidmed2. Has a second piece of fruit every day.	No	7(63.6%)	64(78%)	124(68.9%)	195(71.4%)	0.377
	Yes	4(36.4%)	18(22%)	56(31.1%)	78(28.6%)	
Kidmed3. Has fresh or cooked vegetables regularly once a day.	No	3(27.3%)	28(34.1%)	53(29.4%)	84(30.8%)	0.642
	Yes	8(72.7%)	54(65.9%)	127(70.6%)	189(69.2%)	
Kidmed4. Has fresh or cooked vegetables regularly more than once a day.	No	7(63.6%)	54(65.9%)	120(66.7%)	181(66.3%)	0.829
	Yes	4(36.4%)	28(34.1%)	60(33.3%)	92(33.7%)	
Kidmed5. Consumes fish regularly (at least 2–3/week).	No	4(36.4%)	40(48.8%)	67(37.2%)	111(40.7%)	0.201
	Yes	7(63.6%)	42(51.2%)	113(62.8%)	162(59.3%)	
Kidmed6. Goes >1/week to a fast food restaurant (hamburger chain).	No	8(72.7%)	56(68.3%)	135(75%)	199(72.9%)	0.357
	Yes	3(27.3%)	26(31.7%)	45(25%)	74(27.1%)	
Kidmed7. Likes pulses and eats them >1/week.	No	1(9.1%)	14(17.1%)	39(21.7%)	54(19.8%)	0.218
	Yes	10(90.9%)	68(82.9%)	141(78.3%)	219(80.2%)	
Kidmed8. Consumes pasta or rice almost every day (5 days or more per week).	No	9(81.8%)	67(81.7%)	139(77.2%)	215(78.8%)	0.416
	Yes	2(18.2%)	15(18.3%)	41(22.8%)	58(21.2%)	
Kidmed9. Has cereal or grains (bread, etc.) for breakfast.	No	3(27.3%)	15(18.3%)	40(22.2%)	58(21.2%)	0.774
	Yes	8(72.7%)	67(81.7%)	140(77.8%)	215(78.8%)	
Kidmed10. Has cereal or grains (bread, etc.) for breakfast.	No	8(21.7%)	49(59.8%)	96(53.3%)	153(56%)	0.146
	Yes	3(27.3%)	33(27.3%)	84(46.7%)	120(44%)	
Kidmed11. Uses olive oil at home.	No	0(0%)	1(1.2%)	4(2.2%)	5(1.8%)	0.469
	Yes	11(100%)	81(98.8%)	176(97.8%)	268(98.2%)	
Kidmed12. Skips breakfast.	No	9(81.8%)	76(92.7%)	157(87.2%)	242(88.6%)	0.540
	Yes	2(18.2%)	6(7.3%)	23(12.8%)	31(11.4%)	
Kidmed13. Has a dairy product for breakfast (yogurt, milk, etc.).	No	2(18.2%)	33(40.2%)	71(39.4%)	106(38.8%)	0.456
	Yes	9(81.8%)	49(59.8%)	109(60.6%)	167(61.2%)	
Kidmed14. Has commercially baked goods, or pastries for breakfast.	No	9(81.8%)	76(92.7%)	172(95.6%)	257(94.1%)	0.074
	Yes	2(18.2%)	6(7.3%)	8(4.4%)	16(5.9%)	
Kidmed15. Has 2 yoghurts and/or 40 g of cheese daily.	No	8(71.7%)	57(69.5%)	122(67.8%)	187(68.5%)	0.684
	Yes	3(27.3%)	25(30.5%)	58(32.2%)	86(31.5%)	
Kidmed16. Eats sweets and/or candy several times a day.	No	11(110%)	78(95.1%)	171(95%)	260(95.2%)	0.631
	Yes	0(0%)	4(4.9%)	9(5%)	13(4.8%)	

^a^ Chi-square for linear trend.
